# Neutrophil elastase and its therapeutic effect on leukemia cells

**DOI:** 10.3892/mmr.2015.3946

**Published:** 2015-06-17

**Authors:** KAI-LING JIANG, PENG-PENG MA, XIAO-QUN YANG, LIANG ZHONG, HUI WANG, XIN-YU ZHU, BEI-ZHONG LIU

**Affiliations:** 1 Central Laboratory of Yong-Chuan Hospital, Chongqing Medical University, Chongqing 402160, P.R. China; 2 Key Laboratory of Laboratory Medical Diagnostics, Ministry of Education, Department of Laboratory Medicine, Chongqing Medical University, Chongqing 400016, P.R. China

**Keywords:** neutrophil elastase, leukemia, proliferation, apoptosis, GW311616A

## Abstract

Neutrophil elastase (NE) is an early myeloid-specific serine protease, which is predominantly produced by promyelocytes. A previous study demonstrated that NE has an important role in the development of acute promyelocytic leukemia (APL). The process of APL was shown to be accelerated in animals that expressed abundant NE, whereas NE-deficient mice were protected from APL development; thus suggesting an important role for NE in the development of APL. The present study aimed to investigate the effects and possible mechanisms of NE. Up- and downregulation of NE in various leukemia cell lines was conducted in order to explore its significance in the occurrence and procession of leukemia, with the aim of identifying novel targeted therapeutic drugs for the treatment of leukemia. NE was overexpressed in cells following infection with an adenovirus, and Cell Counting kit-8 and flow cytometry results demonstrated that cell proliferation was promoted, and cell apoptosis was inhibited, as compared with the untreated cells. NE was downregulated in the cells by both RNA interference and treatment with GW311616A, a specific inhibitor of NE, following which cell growth was shown to be inhibited and apoptosis was induced. These results suggested that NE may promote the development of APL, therefore, NE may be a therapeutic target and its inhibitor GW311616A may be a potential therapeutic drug for leukemia. Furthermore, the apoptosis-associated protein B-cell lymphoma 2 (Bcl-2)-associated X protein was significantly increased, whereas Bcl-2 was markedly decreased in the cells with downregulated NE. Further experiments revealed that the probable apoptosis-associated signaling pathway was the phosphoinositide 3-kinase/AKT pathway. The present study is the first, to the best of our knowledge, to demonstrate that GW311616A, a specific NE inhibitor, may act as a potential targeted drug for leukemia, which may have a profound impact on the future of leukemia-targeted therapy.

## Introduction

Neutrophil elastase (NE) is a neutrophil-specific serine protease, which is able to degrade virulence factors and kill bacteria. NE knockout mice are susceptible to bacterial and fungal infections (1,2). It has also been suggested that NE may be involved in the development of obesity and obesity-related complications (3). Since NE has various functions, numerous studies have focused on its effects on inflammatory diseases, such as acute lung injury and obesity (3–5). Previous studies have demonstrated that NE contributes to the progression of various malignancies, and promotes cell proliferation and metastasis during the occurrence and development of certain solid tumors (6,7).

NE is predominantly produced in promyelocytes. Lane and Ley (8) demonstrated that importing the fusion protein, PML-RARα, into early myeloid cells that express high levels of NE resulted in a marked increase in the risk of acute promyelocytic leukemia (APL); therefore, NE expression may aid in determining susceptibility of hematopoietic cells to APL (9). To date, there have been few studies regarding the effects of NE on leukemia; therefore, determining the effect of NE on leukemia may provide evidence for the development of novel target drugs for leukemia therapy.

NE inhibitors, both endogenous and synthetic, are able to inhibit NE activity and regulate the release of inflammatory cytokines and chemokines (10). Endogenous inhibitors include α1-proteinase inhibitor, a secretory leukocyte protease inhibitor and α2-macroglobulin, whereas the synthetic inhibitors include GW311616A and Sivelestat (11,12). GW311616A is a potent, long-acting intracellular inhibitor of human NE. As compared with other inhibitors, it is orally bioavailable, long-lasting and is associated with lower clearance levels (13). Currently, studies regarding NE inhibitors have focused on acute lung injury and chronic obstructive pulmonary disorder (14,15). GW311616A is an NE inhibitor; however, its role in human leukemia has yet to be elucidated. Further studies regarding the proliferation and apoptosis of cells following treatment with NE inhibitors, such as GW311616A, may provide novel information regarding leukemia, and identify a novel direction for future research on leukemia treatment.

In our preliminary experiment, NE expression was detected in five cases of APL (unpublished data), and NE expression was higher in patients with APL, as compared with healthy controls, thus confirming its important role. In order to clarify the role of NE in the process and development of leukemia, two different leukemia cell lines were selected: K562 and U937. The K562 cells express NE at low levels, whereas U937 cells contain abundant NE (8,9).

To explore the possibility of NE as a target for diagnosis and treatment, NE was upregulated in K562 cells by recombinant adenovirus, and silenced in U937 cells by small interfering (si)RNA and treatment with a specific inhibitor of NE, GW311616A. The exact effects of NE were then determined on the various cell lines, including alterations in cell proliferation, apoptosis, expression of apoptotic proteins, and activation of related signaling pathways.

## Materials and methods

### Cell culture and construction of adenovirus (Ad)-NE

The K562 and U937 leukemia cells (Institutes for Biological Sciences, Shanghai, China) were cultured in RPMI 1640 (Gibco Life Technologies, Carlsbad, CA, USA) supplemented with 10% fetal bovine serum (Gibco Life Technologies) at 37°C in an environment containing 5% CO_2_. The medium was refreshed daily. An Ad-Easy system (American Type Culture Collection, Rockville, MA, USA) was used to construct the recombinant adenovirus (16).

### Cell infection by adenovirus

K562 cells (2×10^5^/ml) were cultured in 24-well plates, and were infected with 5 *μ*l recombinant adenovirus Ad-NE (containing NE) and Ad-KZ (empty vector), conjugated to GFP. Fluorescence microscopy was used to observe green fluorescence 48 h post-infection. The cells were divided into three groups: Infection group (K562/Ad-NE), empty vector group (K562/Ad-KZ) and untreated K562 cell group.

### RNA interference

Cell density was 60–80% confluent on the day of transfection. The U937 cells (5×10^5^ logarithmic growth phase cells) were seeded into 6-well plates. For cell transfection, 5 *μ*l siRNA and 5 *μ*l Lipofectamine^®^ 2000 (Invitrogen Life Technologies, Carlsbad, CA, USA) were diluted in 100 *μ*l Opti-MEM (Invitrogen Life Technologies) separately. The siRNA and Lipofectamine^®^ 2000 were then gently mixed and incubated for 25 min at room temperature. The siRNA-Lipofectamine^®^ 2000 complexes were subsequently added to each well and mixed by gentle agitation. All siRNA were purchase from RiboBio (Guangzhou, China) and the sequences of the NE-specific siRNAs were as follows: siRNA101, 5′-CCGUAAACUUGCUCAACGAdTdT-3′ and 3′- dTdTGGCAUUUGAACGAGUUGCU-5′; siRNA102, 5′-CCGGUGGCACAGUUUGUAAdTdT-3′ and 3′-dTdTGGCCACCGUGUCAAACAUU-5′; siRNA103, 5′-GAUCGACUCUAUCAUCCAAdTdT-3′ and 3′-dTdTCU-AGCUGAGAUAGUAGGUU-5′. Following a 48 h incubation at 37°C, red fluorescence protein (RFP) was visualized by fluorescence microscopy. Transfection efficiency was determined by calculating the number of cells expressing RFP. The cells were divided into the following experimental groups: Untreated group, negative control (NC) group, siRNA-101 group, siRNA-102 group and siRNA-103 group.

### NE activity assay

The U937 and K562 cells were seeded into a 6-well plate (6×10^4^ cells/well), and 150 *μ*mol/l GW311616A (Sigma-Aldrich, St. Louis, MO, USA) was subsequently added to the cells. After 48 h, the protein was extracted using radioimmunoprecipitation lysis buffer (Zhongshan Goldenbridge Biotechnology Co., Ltd., Beijing, China) and concentration was measured by a bicinchoninic acid assay (BCA). Purified human leukocyte elastase (Sigma-Aldrich) was used as a standard, and was diluted in pure water to provide the following final concentrations: 100, 200, 400, 800, 1,600 and 3,200 pmol/l. Another three wells contained the protein samples. A total of 100 *μ*l substrate solution (Sigma-Aldrich) was added to the samples and incubated at 37°C for 1 h. The optical density (OD) was measured at 405 nm in an ELISA analyzer. According to the OD value and a linear regression equation of the standards, the activity of NE in the samples was calculated.

### Western blot analysis

The cells (5×10^6^) were collected in Eppendorf tubes, washed with ice-cold phosphate-buffered saline (PBS), and lysed in radioimmunoprecipitation solution containing a protease inhibitor cocktail (Roche, Los Angeles, CA, USA). Protein concentration was determined using the BCA method. A total of 30–50 *μ*g protein was separated by 10% sodium dodecyl sulfate-polyacrylamide gel electrophoresis, and was then transferred to a polyvinylidene difluoride membrane (EMD Millipore, Billerica, MA, USA). The membrane was blocked with 5% skimmed milk (EMD Millipore) for 2 h at room temperature, and was then incubated with the following primary antibodies overnight at 4°C: Mouse anti-human NE monoclonal antibody (diluted 1:2,000; Abcam, Cambridge, MA, USA); and rabbit anti-human polyclonal B-cell lymphoma 2 (Bcl-2), rabbit anti-human polyclonal Bcl-2-associated X protein (Bax) and rabbit anti-human AKT polyclonal antibodies (diluted 1:1,000; Santa Cruz Biotechnology, Inc., Dallas, TX, USA). The membranes were then incubated with goat anti-rabbit and goat anti-mouse secondary antibodies (1:2,000 dilution; Zhongshan Goldenbridge Biotechnology Co., Ltd., Beijing, China) for 1 h at 37°C. After washing with Tris-buffered saline containing Tween-20 (TBST), the immunoreactive complexes were visualized using an enhanced chemiluminescence system (Bio-Rad Laboratories, Inc., Hercules, CA, USA). β-actin was used as an internal positive control.

### Cell Counting kit (CCK)-8 proliferation assay

The cells were plated into 96-well plates (5×10^3^/well). A total of 10 *μ*l CCK-8 was added to the cells, in order to quantify cell proliferation 1 to 4 days post-infection. Following a 2 h incubation, the absorbance of each well was measured at 450 nm, using a spectrophotometer (Bio-Rad Laboratories, Inc.). A cell growth curve was generated with absorbance as the ordinate and time as the abscissa. The experiment was repeated three times.

### Flow cytometric assay

The cells were routinely collected and centrifuged at 500 × g for 5 min at room temperature. After the cells had been washed twice with PBS, a staining mixture was prepared containing 5 *μ*l Annexin-V-fluorescein isothiocyanate fluorescent dye and 5 *μ*l propidium iodide (Sigma-Aldrich). The rate of cell apoptosis was analyzed using a FACsorter (BD Biosciences, San Jose, CA, USA) following a 15 min incubation at room temperature. Furthermore, 5×10^5^ cells were collected, centrifuged at 500 × g for 5min and washed with pre-cooled PBS twice. The supernatant was discarded and precooled 70% ethanol (1 ml) was added and the samples were fixed overnight at 4°C. Following fixation, the samples were centrifuged at 500 × g for 5 min and washed with 1 ml PBS twice. The samples were resuspended cells in 100 *μ*l 1 mg/ml RNase solution in a 37°C water bath for 30 min. Following incubation, 150 *μ*l 50 *μ*g/ml propidium iodide staining solution was added and incubated for 30 min in dark at room temperature. The cell cycle distribution was detected using a FACsorter. Experiments were repeated three times.

### Statistical analysis

Statistical analyses were performed using SPSS 17.0 software (SPSS Inc., Chicago, IL, USA). Experimental results are presented as the mean ± standard deviation. An independent samples t-test was used to compare the results of two groups. P<0.05 was considered to indicate a statistically significant difference. Each experiment was repeated at least three times.

## Results

### NE protein expression levels in K562 cells

**Figure 1:**
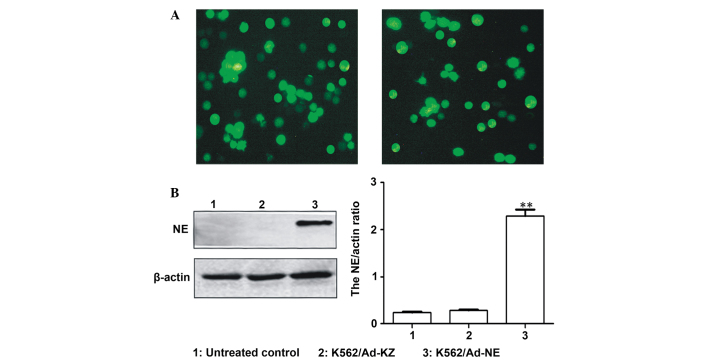
Neutrophil elastase (NE) protein expression in K562 human leukemia cells. (A) Green fluorescent protein expression in infected cells was detected by fluorescence microscopy (magnification, ×200), indicating successful expression of adenovirus containing NE. (B) Detection of NE in the infected K562 cells by western blotting. The protein expression levels of NE were significantly higher in the K562/Ad-NE cells, as compared with the control groups. Data are expressed as the mean ± standard deviation. ^**^P<0.001, compared with the control. Ad, adenovirus.

To upregulate NE gene expression, the K562 cells were infected with recombinant adenovirus Ad-NE or Ad-KZ with enhanced green fluorescent protein (GFP). After 48 h, the percentage of GFP-positive cells in the Ad-NE and Ad-KZ infection groups was 70 and 60%, respectively (Fig. 1A). These results suggest that the K562 cells were successfully infected with the recombinant adenoviruses, as determined by expression of the GFP reporter gene. The protein expression levels of NE were detected following infection of the cells with Ad-NE, by western blotting. The expression levels of NE were significantly higher in the K562/Ad-NE group, as compared with the control groups (P<0.001, Fig. 1B).

### Effects of Ad-NE on proliferation, apoptosis and cell cycle distribution in K562 cells

**Figure 2:**
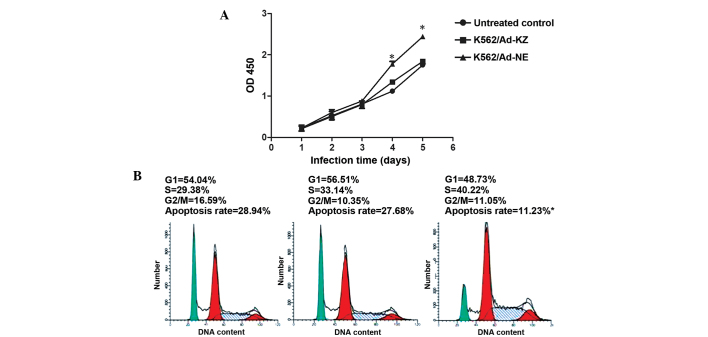
Effects of adenovirus-neutrophil elastase (Ad-NE) on proliferation, apoptosis and cell cycle distribution in K562 human leukemia cells. (A) Cell Counting kit-8 assay was used to measure cell proliferation on days 1, 2, 3, 4 and 5. Absorbance was measured at 450 nm. The proliferation of Ad-NE-infected cells was significantly enhanced in a time-dependent manner. Data are expressed as the mean ± standard deviation. ^*^P<0.05. (B) Flow cytometry was used to detect cell cycle distribution and apoptotic rate. The cell cycle of the Ad-NE infected cells was arrested in S phase. The apoptotic rate of the infected cells was markedly inhibited, as compared with the control cells. ^*^P<0.05, compared with the control. Lane 1: K562 cells; lane 2: K562/Ad-KZ cells; and lane 3: K562/Ad-NE cells.

To determine whether upregulation of NE had an effect on cell growth and apoptosis *in vitro*, CCK-8 and flow cytometric analysis were performed to detect the proliferation and apoptosis of the K562 cells, respectively. The CCK-8 assay showed that cell growth was markedly enhanced in the Ad-NE group in a time-dependent manner, as compared with the untreated control and Ad-KZ groups (P<0.05, Fig. 2A). Flow cytometric analysis demonstrated there was no significant difference in cell cycle distribution; however, the rate of apoptosis was reduced. The apoptotic rate in the Ad-NE group was 11.23±0.56%, whereas the apoptotic rate in the Ad-KZ and blank groups was 28.94±0.44 and 27.68±0.49%, respectively, thus indicating that NE was able to inhibit apoptosis of K562 cells (P<0.05, Fig. 2B).

### Efficiency of siRNA and GW311616A on NE expression in various cell lines

**Figure 3:**
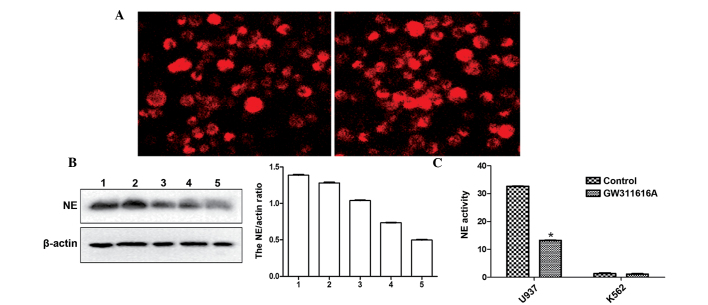
Detection of neutrophil elastase (NE) following downregulation by small interfering (si)RNA and GW311616A in two cell lines. (A) Red fluorescent protein expression was detected in the siRNA-transfected U937 human leukemia cells by fluorescence microscopy (magnification, ×200), indicating successful expression of the siRNA in the U937 cells. (B) Western blotting was performed to measure the protein expression levels of NE in U937 siRNA-transfected cells. The protein expression levels of NE in the 5th group were significantly decreased in the U937/siRNA-103 group, as compared with control groups. Representative results of western blot and densitometric analysis for quantitative evaluation are shown. Data are presented as the mean ± standard deviation of triplicate experiments. ^***^P<0.001, vs. untreated control. Lane 1, U937 cells; lane 2, U937/si negative control (NC) cells; lane 3, U937/siRNA-101 cells; lane 4, U937/siRNA-102 cells; and lane 5, U937/siRNA-103 cells). (C) Effects of GW311616A on NE activity in U937 and K562 human leukemia cells. NE activity was strongly surpressed by GW311616A. ^*^P<0.05, vs. control.

In order to determine the function of NE in leukemia, the present study knocked down its endogenous expression by RNA interference. The percentage of RFP-positive cells in the U937/siRNA group was 85% 48 h post-transfection (Fig. 3A). The effects of RNA interference were also detected by western blotting. The protein expression levels of NE were decreased in the three RNA interference groups, as compared with the untreated and NC groups, and the interference efficiency was most marked in the U937/siRNA-103 group (P<0.001, Fig. 3B). NE activity was tested before and after treatment with GW311616A in two cell lines, and GW311616A was shown to markedly suppress NE activity (Fig. 3C).

### Cell proliferation and apoptosis in U937 cells following knockdown of NE expression

**Figure 4:**
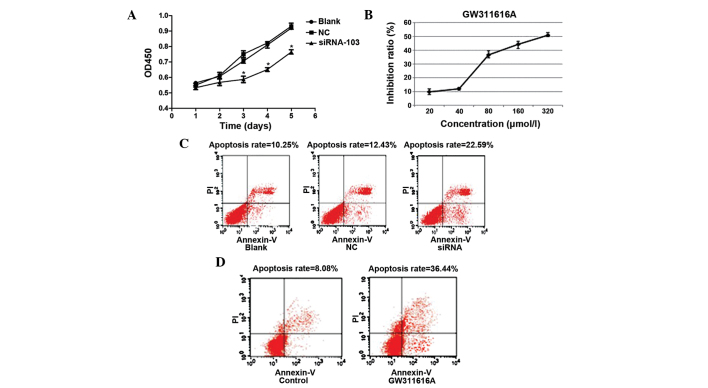
(A) Detection of cell proliferation following neutrophil elastase (NE) knockdown. The proliferation of small interfering (si)RNA-103-infected U937 human leukemia cells was significantly inhibited in a time-dependent manner. Data are expressed as the mean ± standard deviation. ^*^P<0.05, compared with the control. (B) Cell proliferation was inhibited in a dose-dependent manner in U937 cells. (C) Rate of apoptosis in the various groups. (D) Rate of apoptosis both prior to and after GW311616A treatment in U937 cells.

In order to accurately evaluate the effect of NE knockdown, the growth and apoptosis of siRNA-infected and GW311616A-treated U937 cells was detected. Following downregulation of NE expression, cell proliferation was inhibited in a time-dependent manner (Figs. 4A and B). Flow cytometric analysis was performed to measure the rate of apoptosis. The rate of apoptosis was markedly higher in the siRNA-103 group (22.59±0.51%), as compared with the NC siRNA group (12.43±0.62%) and the untreated group (10.25±0.45%) 48 h post-transfection (P<0.05, Fig. 4C).

In the GW311616A-treated group, the rate of apoptosis was enhanced, as compared with the control group (P<0.05, Fig. 4D).

### Expression levels of apoptosis-associated proteins Bax and Bcl-2

**Figure 5:**
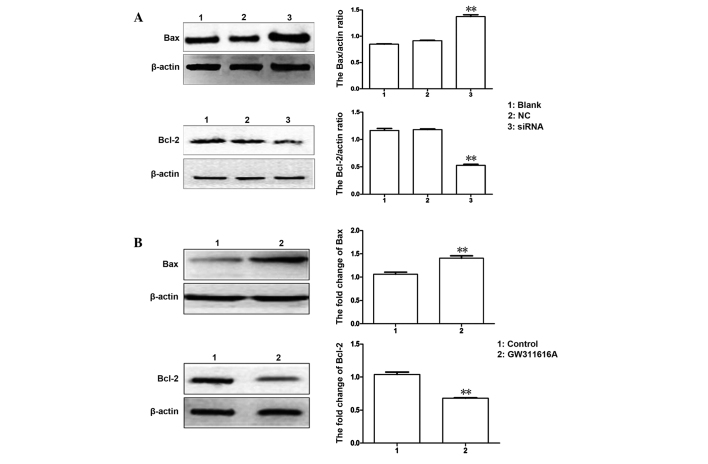
Expression levels of apoptosis-associated proteins B-cell lymphoma 2 (Bcl-2) and Bcl-2-associated X protein (Bax). (A) Western blotting analyzed the protein expression of Bax and Bcl-2 following downregulation of neutrophil elastase (NE) by targeted siRNA-103 in U937 human leukemia cells. ^**^P<0.01. (B) Western blotting detected the protein expression of Bax and Bcl-2 following treatment with GW311616A. ^**^P<0.01, compared with the control. NC, negative control.

To further investigate the effects of NE on apoptosis in leukemia, the expression levels of relevant apoptosis-associated proteins Bax and Bcl-2 were detected following NE knockdown in U937 cells. Downregulation of NE with siRNA-103 was able to increase the protein expression levels of Bax and decrease the expression of Bcl-2, as compared with the control group (Fig. 5A). Following treatment with GW311616A, similar results were observed (Fig. 5B).

### Associated signaling pathway

**Figure 6:**
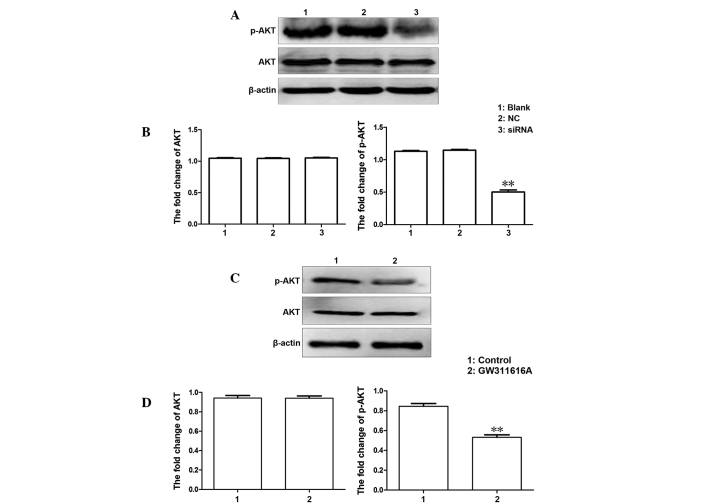
Expression of Akt and phosphorylated (p)-Akt in the various groups. (A) Western blotting was used to analyze the protein expression of AKT and p-AKT in U937 human leukemia cells following transfection with small interfering (si)RNA-103. (B) Quantitative analysis of western blotting. ^**^P<0.01, compared with the control. (C) Expression of AKT and p-AKT in U937 cells following treatment with GW311616A. (D) Quantitative analysis of western blotting. Data are presented as the mean ± standard deviation. NC, negative control.

In order to determine the underlying mechanism of the effects of siRNA and GW311616A on the proliferation and apoptosis of leukemia cells, the expression levels of AKT and phosphorylated-AKT in the PI3K/AKT signaling pathway were detected. The expression levels of phosphorylated AKT were decreased, whereas the expression levels of total AKT were similar in each group, which suggested that the siRNA inhibited the activation of the AKT signaling pathway (Fig. 6).

## Discussion

In recent years, numerous studies have focused on leukemia, particular aims have been exploration of its therapeutic targets and drug interventions, and identification of novel approaches for molecular diagnosis.

NE, encoded by Ela-2, is a type of serine protease (17), which is predominantly expressed in promyelocytes and packaged in azurophilic granules (9). Numerous studies have reported on its involvement in the progression of certain tumors, such as breast cancer, non-small cell lung cancer and colorectal cancer (3,18,19). In addition, through the generation of elastin fragments, NE may potently stimulate cancer cell invasiveness and angiogenesis (20). A previous study identified natural compounds and synthetic agents that are able to antagonize NE activity, which may be of value as therapeutic agents for suppressing cancer cell growth in certain types of cancer (8). The effects of NE in leukemia were initially investigated by Lane *et al* (8,9), whose studies demonstrated the importance of NE in the occurrence and development of APL. Since NE was identified as having an important role in the occurrence of APL, it is of great importance to determine the underlying mechanisms of the effects of NE in leukemia cells, and to detect its effects on leukemia cell lines that express abundant of NE. These findings may be beneficial for the identification of specific inhibitors to treat leukemia through inhibition of NE activity.

In order to explore the exact effects of NE and to clarify its underlying mechanism, the present study upregulated NE in K562 cells, which express little NE; and downregulated NE in U937 cells, which contain various levels of NE. Following up- or downregulation of NE, the proliferation and apoptosis of the two cell lines was measured, and the results demonstrated that NE had a proliferation-inducing and anti-apoptotic effect in leukemia cells. The cell cycle of the Ad-NE infected cells was arrested in S-phase, indicating that NE may be a potential therapeutic target.

It is well-known that apoptosis is characterized by a series of biochemical events, including condensation of the nuclei, DNA fragmentation, and ultimately cell death (21,22). The Bcl-2 protein family contains key regulators of apoptosis, including apoptosis-inducing factors (e.g. Bax) and anti-apoptotic factors (e.g. Bcl-2) (23–25). A slight change in the dynamic balance of anti-apoptotic to pro-apoptotic proteins may result in either inhibition or promotion of cell death (26–28).

The results of the present study demonstrated that silencing NE by siRNA in U937 cells resulted in an upregulation of the expression of Bax, whereas Bcl-2 expression was downregulated. Further experiments demonstrated that these findings may be achieved through regulation of the PI3K/AKT pathway, which has an important role in orchestrating various cellular processes, including proliferation, differentiation and apoptosis (29–31). The expression levels of phosphorylated-AKT were decreased following the downregulation of NE using siRNA, whereas the expression levels of total AKT were similar in each group, thus indicating that the activation of phosphorylated-AKT in the PI3K/AKT pathway was inhibited by siRNA, which induced apoptosis-associated protein Bax and Bcl-2 expression changes. These results demonstrated the positive effects of low level expression of NE.

To further understand the effects of NE, and to identify therapeutic targets for leukemia, a specific inhibitor of NE, GW311616A was selected. GW311616A is a potent, intracellular, orally bioavailable, and long-acting inhibitor of human NE (2,32). In the present study, the inhibitory efficiency of GW311616A was apparent, and its ability to induce apoptosis in a concentration-dependent manner was observed. In the GW311616A-treated group, cell apoptosis was enhanced (P<0.05). These results demonstrated the therapeutic effects of GW311616A. Furthermore, the expression levels of apoptosis-associated proteins Bax and Bcl-2, phosphorylated-AKT and total AKT were detected, the results of which were the same as in the siRNA-treated groups; thus suggesting that GW311616A has a potential therapeutic effect.

In the present study, the protein expression levels of apoptosis-inducing Bax were reduced and the expression levels of anti-apoptotic proteins were increased following downregulation of NE, by siRNA and treatment with GW311616A. These results indicated that NE may be a therapeutic target for leukemia, and the inhibitory drug GW311616A may be used as a potential treatment. However, it will be beneficial to continue studying the effects of NE inhibition in various leukemia cell lines, including HL60 cells.

The present study demonstrated that upregulating NE was able to promote the growth of leukemia cells and decrease the proportion of apoptotic cells. Conversely, downregulation of NE by siRNA and GW311616A treatment inhibited proliferation and induced apoptosis in leukemia cells. This novel information regarding the involvement of NE in leukemia cell proliferation and apoptosis may be used to identify a potential biomarker or treatment target for leukemia, and the NE-specific inhibitor GW311616A may be a promising treatment target option.
